# Performance Analysis of Artificial Neural Network and Its Optimized Models on Compressive Strength Prediction of Recycled Cement Mortar

**DOI:** 10.3390/ma18245694

**Published:** 2025-12-18

**Authors:** Lin-Bin Li, Guang-Ji Yin, Jing-Jing Shao, Ling Miao, Yu-Jie Lang, Jia-Jia Zhu, Shan-Shan Cheng

**Affiliations:** 1 Department of Civil and Transportation Engineering, Ningbo University of Technology, Ningbo 315211, China; 18958880041@163.com (L.-B.L.); yinxjlove@163.com (G.-J.Y.); langyj_jul@163.com (Y.-J.L.); synnabcd@163.com (J.-J.Z.); 2 College of Art and Design, Nanjing Forestry University, Nanjing 210037, China; miaoling@njfu.edu.cn; 3 School of Engineering, Computing and Mathematics, University of Plymouth, Plymouth PL4 8AA, UK; shanshan.cheng@plymouth.ac.uk

**Keywords:** performance analysis, artificial neural network, compressive strength, recycled cement mortar, optimization algorithm

## Abstract

In the background of sustainable development in the construction industry, recycled cement mortar (RCM) has emerged as a research hotspot due to its eco-friendly features, where mechanical properties serve as critical indicators for evaluating its engineering applicability. This study proposes an artificial neural network (ANN) model optimized by intelligent algorithms, including the GWO (grey wolf optimizer), PSO (particle swarm optimization), and a GA (genetic algorithm), to predict the compressive strength of recycled mortar. By integrating experimental and prediction data, we establish a comprehensive database with eight input variables, including the water–cement ratio (W/C), cement–sand ratio (C/S), fly ash content (FA), aggregate replacement rate (ARR), and curing age. The predictive performance of neural network models with different database sizes (database 1: experimental data of RCM; database 2: experimental data of RCM and ordinary mortar; database 3: model prediction data of RCM, experimental data of RCM, and ordinary mortar) is analyzed. The results show that the intelligent optimization algorithms significantly enhance the predictive performance of the ANN model. Among them, the PSO-ANN model demonstrates optimal performance, with *R*^2^ = 0.92, MSE = 0.007, and MAE = 0.0632, followed by the GA-ANN model and the GWO-ANN model. SHAP analysis reveals that the W/C, C/S, and curing age are the key variables influencing the compression strength. Furthermore, the size of the dataset does not significantly influence the computation time for the above models but is primarily governed by the complexity of the optimization algorithms. This study provides an efficient data-driven method for the mix design of RCM and a theoretical support for its engineering applications.

## 1. Introduction

The continuous development of urbanization in China has resulted in a sharp increase in the usage of concrete, but has also generated vast amounts of waste concrete, with statistics indicating that it produces 2 billion tons annually [1]. To achieve sustainable development in the construction industry, an essential solution involves processing waste concrete through separation, crushing, and cleaning to produce recycled aggregates as substitutes for natural aggregates [2]. However, residual old cement paste on the surface of recycled aggregates and new micro-cracks formed during crushing result in inferior performance compared with natural aggregates. After substitution, the overall performance of cement-based materials significantly declines [3]. Leite et al. [4] found that concrete prepared with recycled coarse aggregates under dry conditions exhibits notably larger pores. Yu et al. [5] observed that maintaining mechanical performance after replacing natural aggregates requires a substantial reduction in workability, making it difficult to meet engineering demands. Therefore, the study by Zhang et al. [6] indicated that the shortcomings introduced by recycled aggregates can be effectively mitigated through the use of enhanced recycled mortar, which leads to improvements in compressive, splitting tensile, and flexural strengths. Kisku et al. [7] prepared recycled coarse aggregate concrete by using the equivalent mortar volume method and a two-stage mixing approach. The results show that the cement hydration enhances, and the porosity reduces, resulting in the mechanical property closely matching that of natural aggregate concrete.

In addition to recycled coarse aggregate, recycled fine aggregate (with a particle size of less than 4.75 mm) is also the main product of waste concrete, accounting for 40–60% of the total mass. Utilizing recycled fine aggregates to produce RCM is a reliable resource conversion pathway [8,9,10]. However, recycled fine aggregates contain more residual old mortar compared with recycled coarse aggregates, so more factors need to be considered for preparing RCM, resulting in extensive mix proportion experiments to explore its performance. Cheng et al. [11] investigated the impact of ARR and found that as the ARR increases, the flowability of RCM reduces by 0.95–10.00%, and the compressive and flexural strengths generally decrease, but the toughness improves. Conversely, Li et al. [12] adopted graded replacement by particle size, demonstrating that the compressive strength of RCM increases by 59.4–87.3% compared with that of ordinary cement mortar (OCM) as the ARR rises. Many scholars optimize RCM performance by incorporating mineral admixtures. Through orthogonal experiments, Fan et al. [13] identified an optimal mix, namely, 70% ARR and 10% FA, as a substitute for cement. The research of Li et al. [14] showed that the addition of FA can reduce the drying shrinkage of RCM, while silica fume (SF) enhances the early strength, and slag (SG) can improve the flowability. Thus, a rational mix design is critical for ensuring RCM performance. Practical engineering requires extensive mix proportion experiments to determine precise parameter ranges, balancing the flowability and compressive strength of RCM while meeting application requirements. Nevertheless, this intricate design process hinders the widespread adoption of RCM [15].

In recent years, artificial intelligence (AI) technologies have gained extensive application in predicting the performance of cement-based materials, gradually replacing empirical formulas [16,17]. Compared with conventional methods, machine learning offers advantages such as strong data-driven capabilities and low dependence on complex mathematical formulas [18,19], enabling autonomous learning from historical data to establish high-precision prediction models. In the research on cement-based materials, different neural network models exhibit distinct strengths. Convolutional neural networks (CNNs) have powerful image feature extraction capabilities and are widely used for characterizing and analyzing material microstructures [20]. Recurrent neural networks (RNNs) are frequently employed for durability and service life prediction of concrete owing to their advantages in processing sequential data. In addition, various models, including linear regression [21], support vector regression [22], random forests [23], etc., have been explored to predict the mechanical properties of concrete. Among these, ANNs have become a research hotspot due to their simple structure and strong adaptability. The studies of [24,25,26,27,28,29] demonstrate the successful application of ANNs in predicting mechanical properties and carbonation depth for machine-made sand concrete [30], self-compacting concrete [31], recycled concrete [32], and other cement-based materials. However, ANNs suffer from inherent limitations, such as unidirectional gradient descent and being easily trapped in local optima [33]. To address these issues, researchers have proposed traditional optimization algorithms, like a GA [34], PSO [35], differential evolution [36], and bio-inspired algorithms like the GWO [37,38] and the firefly algorithm [39]. These approaches can significantly enhance model convergence speed and global search capabilities.

**Figure 1:**
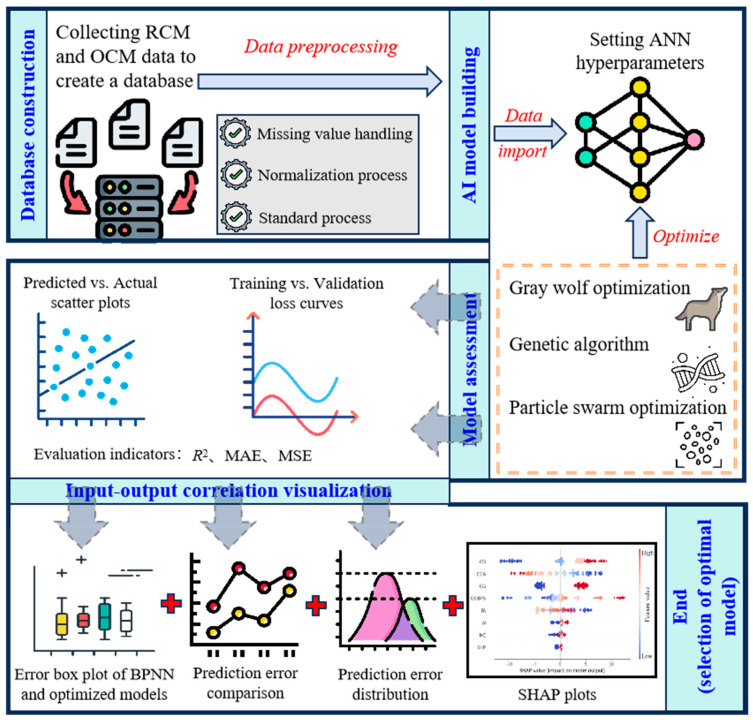
Research approach of this article.

To summarize, utilizing mineral admixtures and recycled aggregates for producing cement-based materials can reduce demand for natural aggregates and cement. Meanwhile, employing machine learning techniques to predict the performance of cement-based materials serves as an effective alternative to complex mix proportion experiments. Although numerous studies have explored neural network prediction models for the mechanical properties of concrete, scant attention has been paid to RCM, with limited analysis of the effect of data volume on model performance. Therefore, this study investigates the performance of an ANN and its optimized models for predicting the compressive strength of RCM on databases with different scales. Through a comprehensive literature review, experimental data on the compressive strength of RCM and OCM are collected, along with RCM compressive strength values predicted by neural network models. This enables the construction of databases with different data volumes. Concurrently, ANN models enhanced by intelligent algorithms, including the GWO, PSO, and a GA, are established for model training and validation. Visualization techniques, such as SHAP plots, training and validation loss curves, and predicted vs. actual value scatter plots of compressive strength, are employed to present the influence of input variables on compressive strength (output), further determining an optimal ANN model. The research approach of this article is shown in Figure 1.

## 2. Methodology

### 2.1. Database

This study generated a comprehensive dataset of input parameters from the existing literature (as shown in Table 1), including 260 sets of experimental data on RCM, 306 sets of experimental data on OCM, and 45 sets of model prediction data on RCM. Each set of data contained eight input variables, including W/C, C/S, water-reducing agent content (WR), FA, SF, SG, ARR, and age, and one output variable, namely, compression strength (CS). To analyze the impact of data types on the predictive performance of ANN models (as shown in Table 2), the dataset was divided into three databases, namely, database 1 (experimental data on RCM), database 2 (experimental data on RCM + OCM), and database 3 (experimental data on RCM + OCM and model prediction data on RCM). The data distribution in different databases can be reflected through the minimum, maximum, average, and 25%, 50%, and 70% quantiles of the data.

### 2.2. Data Preprocessing

**Figure 2:**
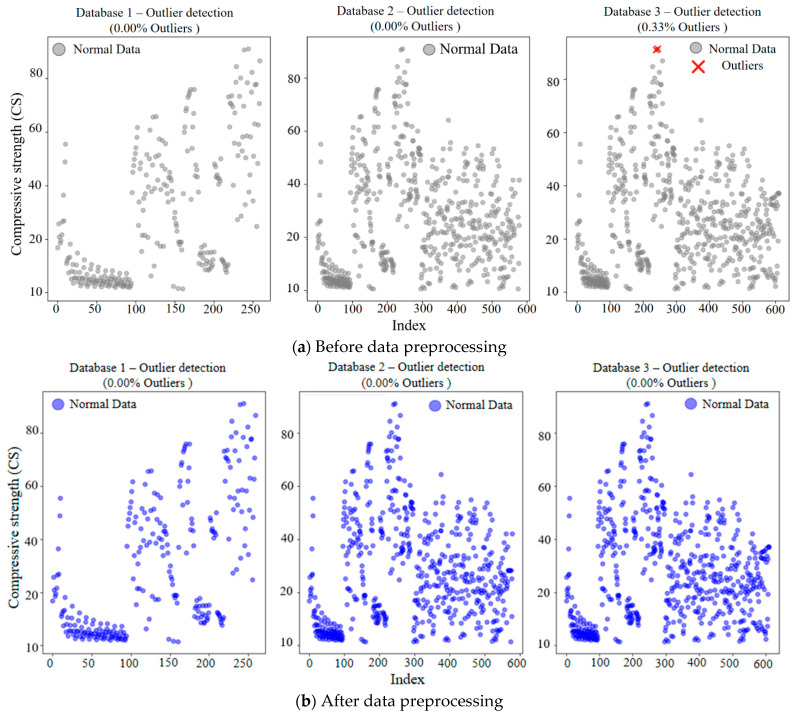
Scatter plot for anomaly detection of data.

The performance of a machine learning model is highly dependent on the data quality of the database. Before model training, it is necessary to systematically evaluate and preprocess the rationality of the dataset to eliminate the influence of outliers caused by data discontinuity. Deleting the outliers directly will reduce the sample size of the data and may lead to model bias. So, we always use estimated values to replace outliers. Common valuation method includes the mean imputation, K-nearest neighbor imputation, and regression imputation. In this paper, the mean imputation is adopted for data preprocessing, which has the advantages of simple calculation and high processing efficiency. The specific calculation formula is shown in Equation (1), and the results of data preprocessing are presented in Figure 2. It can be seen from this figure that the introduction of experimental data on OCM not only significantly increases the sample size of the data but also has good compatibility with experimental data on RCM. The introduction of model prediction data on RCM results in some outliers. After data preprocessing, abnormal data situations are resolved, ensuring the training effectiveness of the model.

(1) $$x_{i}=\left\{\begin{array}{cc} x_{i}, & \;\mathrm{if}\;x_{i}\;\mathrm{is}\;\mathrm{not}\;\mathrm{an}\;\mathrm{outlier}\\ \frac{1}{n}\sum_{j=1}^{n}x_{j}, & \mathrm{if}\;x_{i}\;\mathrm{is}\;\mathrm{an}\;\mathrm{outlier} \end{array}\right.$$

where $x_{i}$ is the *i*-th data in the database, *n* represents the number of outliers, and $\sum_{j=1}^{n}x_{j}$ is the value sum of the outliers.

### 2.3. Data Visualization

**Figure 3:**
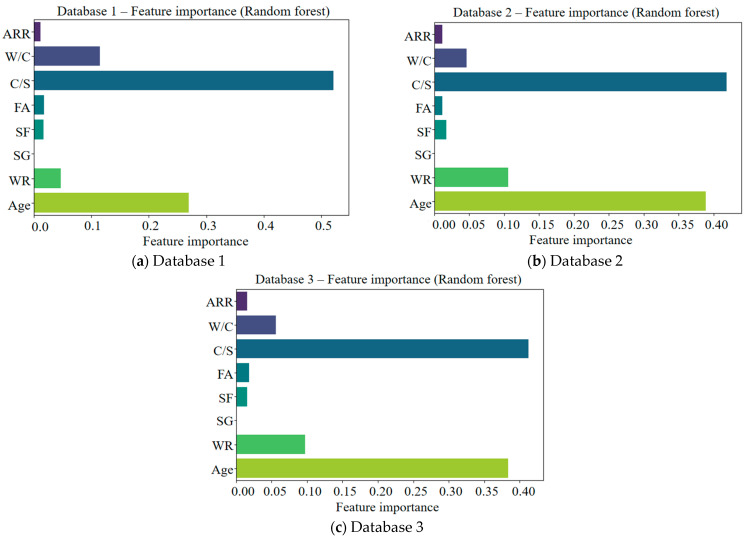
Quantitative evaluation of feature importance of variables for different databases.

The variable importance assessment and correlation analysis between variables are crucial steps of the model training. After data preprocessing, this study used a random forest algorithm to quantitatively evaluate the importance of the variables, and the result is shown in Figure 3. It can be seen from this figure that C/S showed significant importance in the model training, but SG had a lower contribution to the model training. This may have been due to the high correlation between SG and other variables, leading the random forest algorithm to underestimate the potential impact of SG. Therefore, it was necessary to visualize the relationships between different variables.

**Figure 4:**
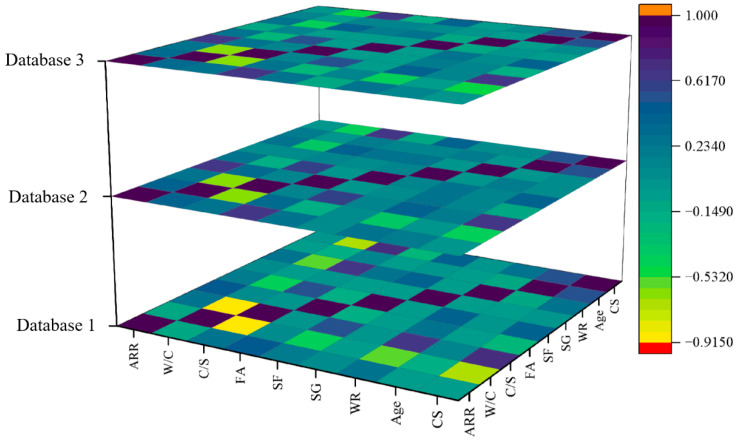
Three-dimensional feature heatmaps of different databases.

Figure 4 presents 3D feature heatmaps visually displaying the variable correlation through the linear strength and direction between variables. As shown in Figure 4, the correlation coefficients between SG and other variables do not exceed 0.2, which is consistent with the results of the random forest analysis mentioned earlier. It can also be seen that CS shows a strong negative correlation with W/C and FA (the correlation coefficients are −0.78 and −0.65), and a strong positive correlation with C/S, SF, and age (the correlation coefficients are 0.82, 0.71, and 0.68). However, the correlation coefficients between AAR, SG, WR, and CS are close to 0, indicating their weak correlation. In addition, as the sample size increases, the correlation coefficients between variables tend to decrease. This can improve the model stability and avoid overfitting and reduced interpretability.

**Figure 5:**
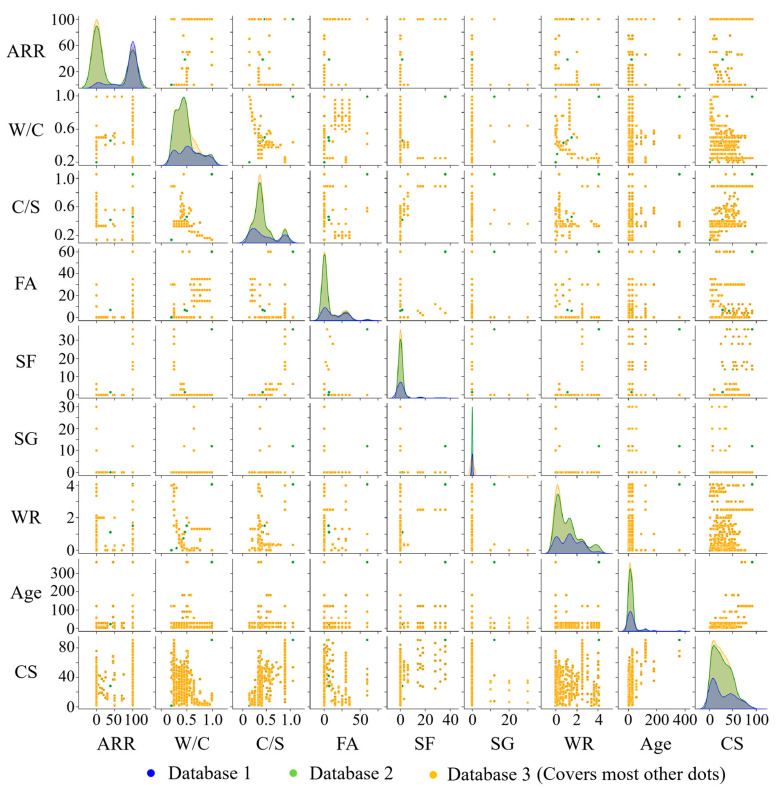
Scatter matrix diagrams for different databases.

Figure 5 is a scatter matrix diagram that reveals the relationship between the input variables and CS, making their correlations more intuitive. The density plot in this figure can visualize the distribution of input variables. As shown in Figure 5, the distribution of W/C data of database 1 (experimental data on RCM) is relatively uniform. With the addition of OCM data (database 2), the amount of data on W/C within 0.5 increases. This indicates that the W/C of OCM is usually lower than 0.5, and its compressive strength is higher than 50 MPa. This indicates that most of the data for ordinary mortar falls within this range, and its compressive strength is higher than 50 MPa. This is because the porous structure of recycled fine aggregate leads to high water absorption. As the ARR reaches 100%, a W/C greater than 0.5 is required to prepare RCM. So, the experimental data of OCM can effectively fill the data gap of the low W/C in database 1.

The distribution pattern of C/S data is similar to that of W/C data. This is because the preparation of RCM needs to improve the C/S (increase the cement proportion to compensate for the strength loss). So, the experimental data of OCM can also effectively fill the data gap of a low C/S. It can also be seen from Figure 5 that the contents of mineral admixtures such as FA, SF, and SG in OCM are all below 50%, while the FA content in RCM is relatively high. This is because FA provides the effect of ball lubrication in RCM, effectively reducing the W/C and further improving its compressive strength. In addition, adding a water-reducing agent is a common method for optimizing the design of RCM, and its content does not exceed 4% of the cementitious material.

### 2.4. Data Scaling and Partitioning

As presented in the data visualization, the numerical ranges of different input variables vary greatly. The input variables with excessively large values dominate the prediction trend of the model, resulting in the overfitting of the model. So, it is necessary to scale and unify the data before the model training, which can accelerate the convergence speed of the model and improve the prediction performance and accuracy. In this paper, the combination of normalization and standardization methods is adopted to scale the data. The normalization method is used to scale all data to between 0 and 1, as shown in Equation (2). The standardization method is used to transform the data into a normal distribution, with an average value of 0 and a standard deviation of 1, as shown in Equations (3) and (4).

(2) $$x_{\mathrm{nd}}=\frac{x-\min\left(x\right)}{\max\left(x\right)-\min\left(x\right)\;}$$

in which $x_{nd}$ is the normalized data, *x* is the raw data, and min(x) and max(x) are the minimum and maximum of the raw data, respectively.

(3) $$x_{\mathrm{sd}}=\frac{x-\mathrm{mean}\left(x\right)}{\mathrm{std}\left(x\right)}$$

(4) $$\mathrm{mean}\left(x\right)=\frac{1}{n}\sum_{i=1}^{n}x_{i},\;\mathrm{std}\left(x\right)=\sqrt{\frac{1}{n}\sum_{i=1}^{n}{\left[x_{i}-\mathrm{mean}\left(x\right)\right]}^{2}}$$

where $x_{\mathrm{sd}}$ is the standardized data, $\mathrm{mean}\left(x\right)$ is the average value of the raw data, $\mathrm{std}\left(x\right)$ is the standard value of the raw data, *n* is the number of raw data, and $x_{i}$ is the *i*-th data of the raw data.

After the data normalization, the data in each database is randomly divided into two groups, with 70% of the data used as the training set and 30% of the data used as the validation set. This random allocation method ensures that both sets of data retain their overall data characteristics and that the model does not lean toward any particular dataset.

## 3. Model and Algorithm

### 3.1. ANN Model

**Figure 6:**
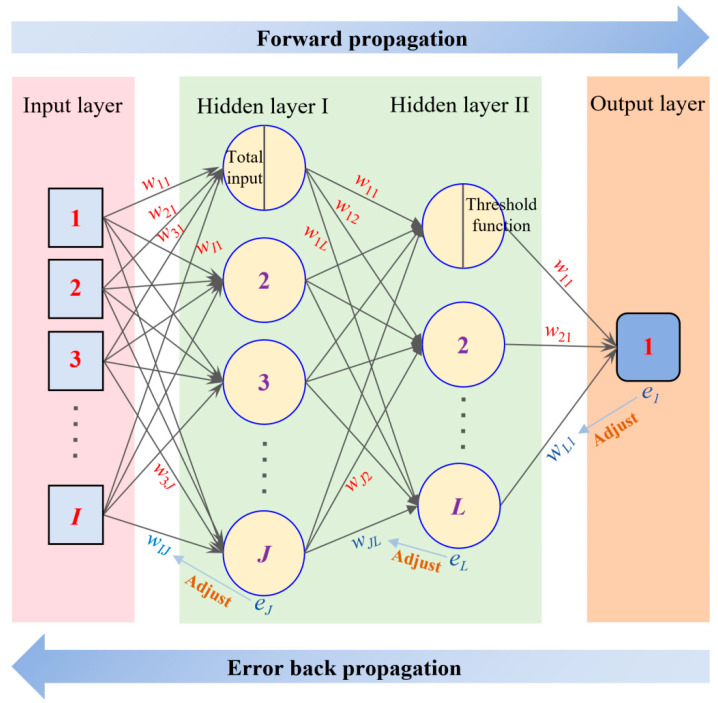
ANN model with 2 hidden layers.

ANNs belong to feedback-type deep neural networks, which are computational models that simulate the working mode of the human brain’s nervous system. In this paper, the topology structure of the ANN is 8-64-32-1 (input layer–hidden layer I–hidden layer II–output layer). The input layer consists of the W/C, C/S, WR, FA, SF, SG, ARR, and age of cement mortar, and the output layer is its CS. As shown in Figure 6, the operation process of the ANN model can be divided into two stages. The first stage is the forward propagation of the signal, which passes through the hidden layer from the input layer and finally reaches the output layer, and the mathematical formula can be expressed as

(5) $$\left\{\begin{array}{l} H_{j}^{I}\left(x_{i}\right)=f\left(\sum_{i=1}^{I}w_{ij}\cdot x_{i}+\theta_{j}\right),\;j=1,\;2,\;\ldots ,\;J\\ H_{l}^{\mathrm{II}}\left(H_{j}^{1}\right)=f\left(\sum_{j=1}^{J}w_{jl}\cdot H_{j}^{I}+\theta_{l}\right),\;l=1,\;2,\;\ldots ,\;L\\ O_{m}\left(H_{l}^{\mathrm{II}}\right)=\sum_{l=1}^{L}w_{lm}\cdot H_{l}^{I}+\theta_{m},\;m=1 \end{array}\right.$$

where *I*, *J*, and *L* represent the number of neurons in the input layer, hidden layer Ⅰ, and hidden layer Ⅱ. $x_{i}$ is the value of the *i*-th neuron in the input layer. $H_{j}^{I}$ is the output value of the *j*-th neuron in the hidden layer Ⅰ, which serves as the input value for the hidden layer Ⅱ after the processing of the activation function $f\left(a\right)$ and bias θ. $H_{l}^{\mathrm{II}}$ is the output value of the *l*-th neuron in the hidden layer Ⅱ. The sigmoid function is often used as an activation function, expressed as Equation (6).

(6) $$f\left(a\right)=\frac{1}{1+e^{\left(\text{-}a\right)}},\;\partial f\left(a\right)/\partial a=f\left(a\right)\cdot \left[1-f\left(a\right)\right]$$

The second stage is the backpropagation of errors. When the error *e_m_* between the result of the output layer (*O_m_*) and the target value (*P_m_*) is large, the weight (*w*) and bias (*θ*) need to be adjusted from the output layer to the input layer sequentially. Its mathematical expression is shown in Equation (7). When the error *e_m_* is less than the set value (*e*_0_), or the maximum number of iterations is reached, the above neural network stops running; otherwise, it returns to the first stage. In short, the ANN model is very sensitive to initial weights and biases [49] and is prone to falling into the dilemma of local minima. So, the chosen initial weights and biases determine the computational efficiency and accuracy of the model.

(7) $$\left\{\begin{array}{l} w_{lm}^{\prime }=w_{lm}-\eta \frac{\partial E}{\partial w_{lm}},\;w_{jl}^{\prime }=w_{jl}-\eta \frac{\partial E}{\partial w_{jl}},\;w_{ij}^{\prime }=w_{ij}-\eta \frac{\partial E}{\partial w_{ij}}\\ \theta_{m}^{\prime }=\theta_{m}-\eta \frac{\partial E}{\partial \theta_{m}},\;\theta_{l}^{\prime }=\theta_{l}-\eta \frac{\partial E}{\partial \theta_{l}},\;\theta_{j}^{\prime }=\theta_{j}-\eta \frac{\partial E}{\partial \theta_{j}}\\ e_{m}=\frac{1}{2}{\left(P_{m}-O_{m}\right)}^{2} \end{array}\right.$$

### 3.2. GWO

**Figure 7:**
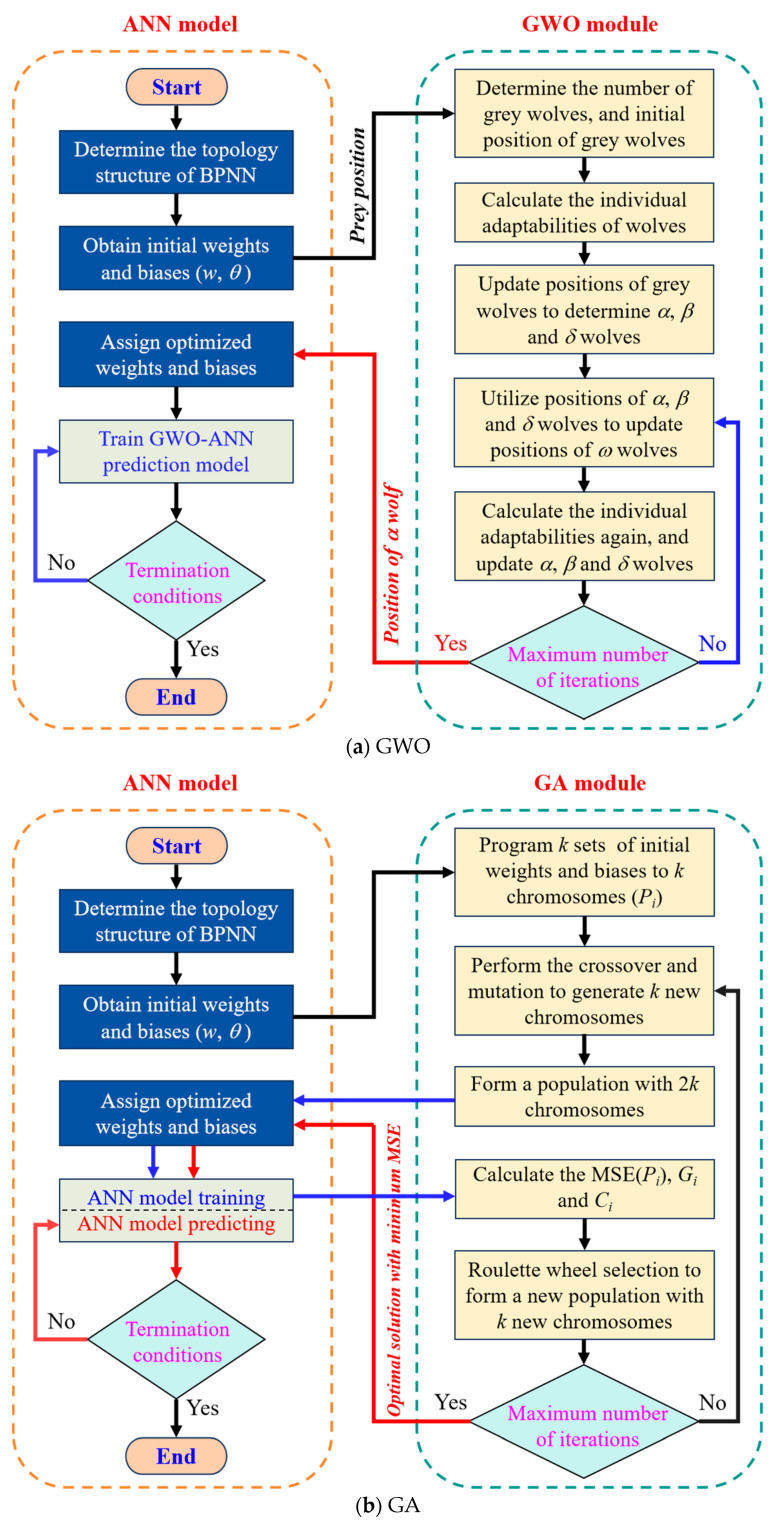

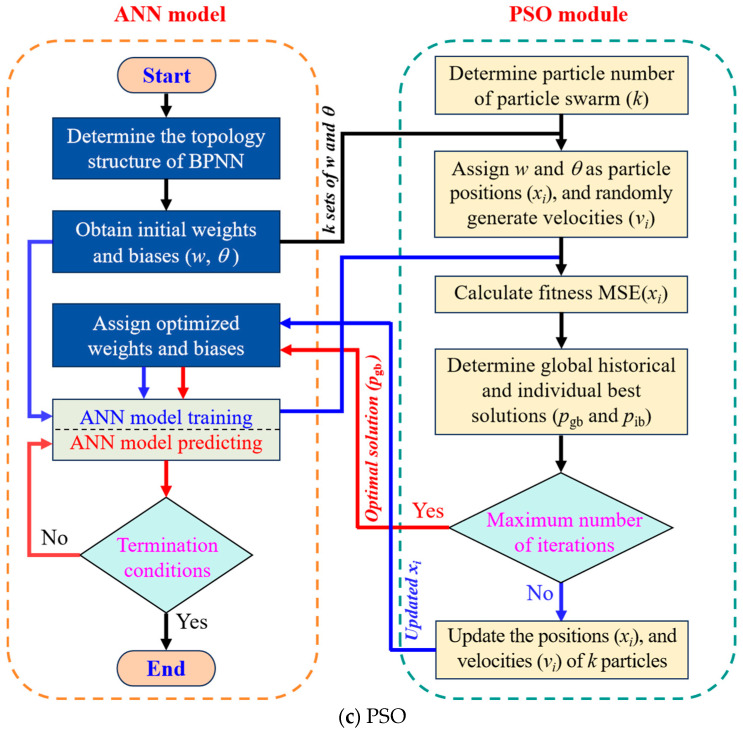
Flowchart of ANN and its optimized models.

The GWO is a global optimization algorithm that helps avoid the local convergence problem of ANN models. It simulates the leadership hierarchy of grey wolves, where *α*, *β*, and *δ* wolves guide the search, and *ω* wolves follow. During the iterative hunting process, the wolf with the best fitness becomes the *α* wolf, representing the current global optimum. Therefore, using GWO to optimize an ANN means updating its weights and biases based on the wolf pack’s search behavior. The overall process is shown in Figure 7a. Firstly, the number of grey wolves (*n*) is determined, and *n* sets of initial weights and biases are randomly generated as the position coordinates of *n* grey wolves. Meanwhile, the initial weights and biases of the ANN model are used as the coordinates of prey. The surrounding algorithm constructed by Mirjalili et al. [38], expressed as Equations (8) and (9), is used to update the coordinates of the grey wolves.

(8) $$D^{t}=\left|C\cdot X_{p}^{t}-X^{t}\right|,\;X^{t+1}=X_{p}^{t}-AD^{t}$$

(9) $$A=2a\cdot r_{1}-a,\;C=2r_{2},\;a=2\cdot \left(1-\frac{t}{M}\right)$$

where *t* is the current iteration. *M* is the maximum number of iterations. $X_{p}^{t}$ is the position (vector) of the prey (the initial position vector obtained by the ANN model, i.e., the optimization objective). $X^{t+1}$ represents the position (vector) of the gray wolf after *t* + 1 times updates. *D* represents the distance between the grey wolf and its prey. *r*_1_ and *r*_2_ are the random vectors generated in [0, 1]. *a* is a linear convergence factor (scalar), while it expands into a vector when it is used to calculate the *A* vector. *C* is a random vector between [0, 2], representing the random weight of the influence of the gray wolf position on the prey position to enhance the algorithm’s global search ability and robustness [53].

**Figure 8:**
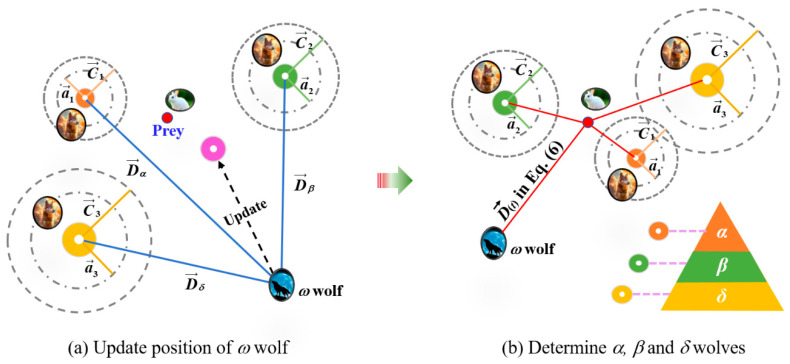
GWO diagram.

Through the calculation of the surrounding algorithm, the positions of wolves can be obtained. The three optimal solutions are selected to become the positions of *α*, *β*, and *δ* wolves, which have potential location information on the prey and can guide *ω* wolves to search for prey, that is, update the positions of *ω* wolves, as shown in Figure 8. The above process can be expressed as Equations (10) and (11). Substituting $X^{t+1}$, obtained from the hunting algorithm of Equation (11), into the surrounding algorithms of Equations (8) and (9), a new round of iteration is conducted to update the positions of $X_{\alpha }$, $X_{\beta }$, and $X_{\delta }$, and then update the position of $X_{\omega }$. Through the continuous iteration, the specified number of iteration times is reached, and the latest position of the α wolf ($X_{\alpha }$) is taken as the optimal solution, which contains the optimized initial weights and biases in the ANN model.

(10) $$D_{\alpha }=\left|C_{1}\cdot X_{\alpha }-X_{\omega }\right|;\;D_{\beta }=\left|C_{2}\cdot X_{\beta }-X_{\omega }\right|;\;D_{\delta }=\left|C_{3}\cdot X_{\delta }-X_{\omega }\right|$$

(11) $$X_{\omega }^{t+1}=\frac{X_{1}+X_{2}+X_{3}}{3};\;X_{1}=X_{\alpha }-A_{1}\cdot D_{\alpha };\;X_{2}=X_{\beta }-A_{2}\cdot D_{\beta };\;X_{3}=X_{\delta }-A_{3}\cdot D_{\delta }$$

in which $X_{\alpha }$, $X_{\beta }$, $X_{\delta }$, and $X_{\omega }$ are the position vectors of *a*, *b*, *δ*, and *ω* wolves.

Based on the above theory, an ANN model optimized by GWO, namely, the GWO-ANN model, is developed to conduct predictive training on the compressive strength of cement mortar. In GWO, 5 gray wolves and 200 iterations are set to optimize the initial weights and biases of the ANN to avoid the local minimum dilemma.

### 3.3. GA

GA is a global optimization algorithm inspired by natural selection and genetic mechanisms, and it can search for optimal solutions in a large solution space. In this study, the initial weights and biases of the ANN model are encoded as chromosome gene segments. Through selection, crossover, and mutation operations, GA updates these parameters and enhances the model’s generalization ability [54]. Compared with the GWO, the GA has higher global search capability, but its computational complexity is higher, and its convergence speed is slower.

**Figure 9:**
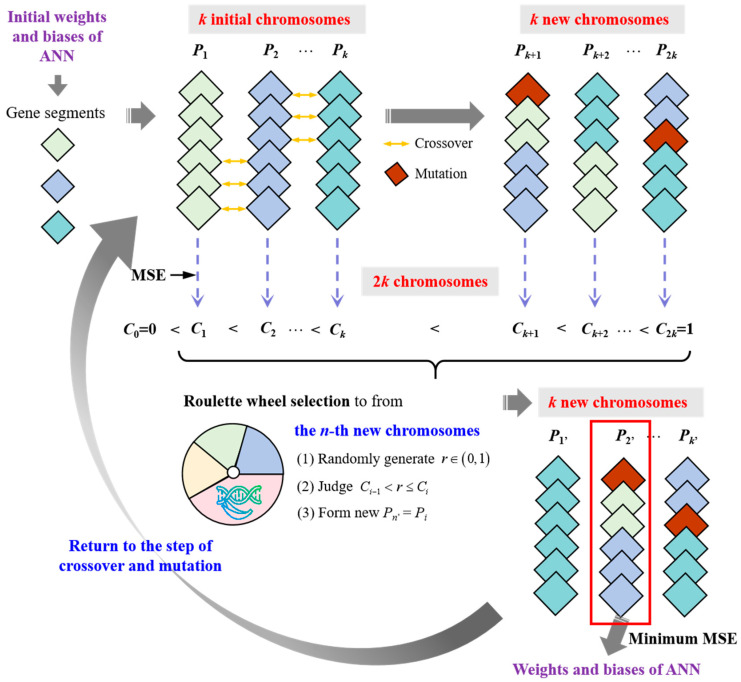
GA schematic diagram.

The GA’s structure is shown in Figure 9. Firstly, the number of chromosomes (*k*) in a population is determined, and the ANN model randomly generates *k* sets of initial weights and biases as the gene segment information of *k* chromosomes, with each chromosome marked as *P_i_* (*i* = 1, 2, …, *k*). Then, crossover recombination is performed on the chromosomes to generate k new chromosomes. Taking *P*_1_ and *P*_2_ as examples, the crossover operation can be expressed mathematically as Equation (12).

(12) $$P_{k+1}=\left\{\begin{array}{c} P_{1}\left(w,\theta \right),\;j\leq c\\ P_{2}\left(w,\theta \right),\;j>c \end{array}\right.;\;P_{k+2}=\left\{\begin{array}{c} P_{2}\left(w,\theta \right),\;j\leq c\\ P_{1}\left(w,\theta \right),\;j>c \end{array}\right.$$

where *j* is the sum of gene segments (weights and biases) in chromosome *P_i_*. *c* is a random number between 1 and *j* − 1. $P_{k+1}^{t}$ and $P_{k+2}^{t}$ are the new chromosomes.

Secondly, the gene mutation is performed on the generated new chromosomes ($P_{k+1}$, $P_{k+2}$, …, $P_{2k}$) by randomly altering certain gene segments to update the new chromosomes, expressed as Equation (13) (taking $P_{k+1}$ as an example). Mutation operations increase the diversity of the population and help avoid getting stuck in local optima. So, a population with 2*k* chromosomes ($P_{1}$, $P_{2}$, …, $P_{2k}$) is formed.

(13) $$p_{k+1}=\left\{\begin{array}{cc} p_{k+1}+\sigma \cdot N\left(0,\;1\right), & r\leq m_{u}\\ p_{k+1}, & r>m_{u} \end{array}\right.;\;p_{k+1}=\left(w_{ij},\;\theta \right)$$

where $p_{1}$ and $p_{k+1}$ are the gene segments in the chromosomes of $P_{1}$ and $P_{k+1}$. *r* is a random number between 0 and 1. *m*_u_ is the mutation probability and is always set as 0.01. σ is the variation amplitude and is usually set to 0.1. $N\left(0,\;1\right)$ is a normal distribution with a mean of 0 and a standard deviation of 1.

Thirdly, the 2*k* chromosomes ($P_{1}$, $P_{2}$, …, $P_{2k}$) with 2*k* sets of weights and biases are introduced into the ANN model to predict the compressive strength. For each chromosome, the output result of predicted compressive strength, marked as *CS*_out_, is compared with the input results of *CS*_data_ to evaluate the fitness of this chromosome by using the mean square error (MSE), as shown in Equation (14). According to the MSE of each chromosome, the *k* times of roulette-wheel selection are performed on the 2*k* chromosomes to form a new population with *k* chromosomes ($P_{1^{\prime }}$, $P_{2^{\prime }}$, …, $P_{k^{\prime }}$). The mathematical expression for roulette-wheel selection is written as Equations (15)–(17).

(14) $$\mathrm{MSE}\left(P_{i}\right)=\frac{1}{M}\sum_{m=1}^{M}{\left(CS_{\mathrm{data}\text{-}m}-CS_{\mathrm{out}\text{-}m}^{i}\right)}^{2}$$

(15) $$G_{i}=\frac{\mathrm{MSE}\left(P_{i}\right)}{{\mathrm{MSE}}_{\mathrm{sum}}},\;{\mathrm{MSE}}_{\mathrm{sum}}=\sum_{i=1}^{2k}\mathrm{MSE}\left(P_{i}\right)$$

(16) $$C_{i}=\sum_{a=1}^{i}G_{a},\;i=\left(1,\;2,\;\ldots ,\;2k\right)$$

(17) $$P_{n^{\prime }\;\left(n=1,\;2,\;\ldots ,\;k\right)}=P_{i},\;\mathrm{if}\;C_{i-1}<r\leq C_{i}$$

where *M* is the sum of datasets in the database. $\mathrm{MSE}\left(P_{i}\right)$ is the mean square error of the *i*-th chromosome $P_{i}$. $CS_{\mathrm{out}\text{-}m}^{i}$ is the output result of CS from the model for the *i*-th chromosome $P_{i}$. $CS_{\mathrm{data}\text{-}m}$ is the date of CS in the database. ${\mathrm{MSE}}_{\mathrm{sum}}$ is the sum of MSE of 2*k* chromosomes ($P_{1}$, $P_{2}$, …, $P_{2k}$). *G_i_* represents the probability of *P_i_* being selected. $C_{i}$ represents the cumulative probability of *P*_1_ to *P_i_* being selected, and *C*_0_ is added, which is equal to 0. *r* is still a random number between 0 and 1. $P_{n^{\prime }\;}\left(n=1,\;2,\;\ldots ,\;k\right)$ is the *n*-th chromosome in the new population.

Then, the crossover, mutation, and selection operations are repeated until the number of iterations reaches the set value, and a new population with *k* chromosomes is obtained. Finally, the chromosome with the best fitness, namely, the minimum MSE, is considered the optimal solution for the weights and biases of the ANN model.

Based on the above theory, an ANN model optimized by a GA, namely, the GA-ANN model, is developed to conduct the prediction of compressive strength of cement mortar. In the GA, the number of chromosomes in a population is set as 20, the iteration number is 50, the mutation probability (*m*_u_) is 0.05, and the variation amplitude (σ) is 0.1, to optimize the initial weights and biases of the ANN. The flowchart of GA optimization for the BNPP model is shown in Figure 7b.

### 3.4. PSO

**Figure 10:**
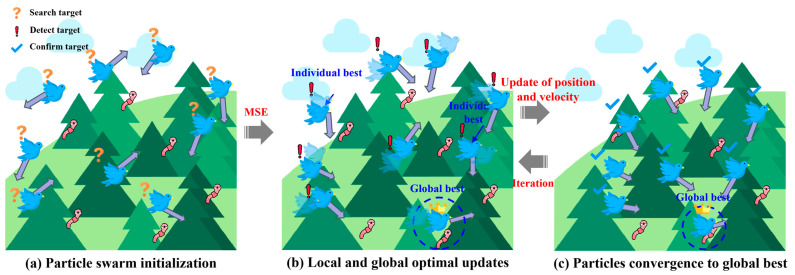
Schematic diagram of PSO structure.

PSO is a stochastic optimization algorithm based on swarm intelligence, inspired by the social behavior of birds and fish, where individuals cooperate and share information to search for optimal solutions. PSO features fast convergence and performs well when the model complexity is moderate. As shown in Figure 10, birds correspond to particles in the swarm, and the forest represents the solution space. The particle search process includes swarm initialization, updating local and global optima, and convergence toward the global best. The core of the PSO algorithm lies in updating particle velocity and position [55].

Figure 7c presents the flowchart of the PSO-optimized ANN (PSO-ANN) model. Firstly, the number of particles (*k*) in a particle swarm is determined, and the ANN model randomly generates *k* sets of initial weights and biases as the positions of *k* particles, marked as *x_i_* (*i* = 1, 2, …, *k*). Meanwhile, the *k* sets of velocity are randomly generated and assigned to *k* particles. For each particle, the position parameter is imported into the ANN model, and the forward propagation operation is performed based on the training set data (without backpropagation) to calculate its fitness, namely, $\mathrm{MSE}\left(x_{i}\right)$. Secondly, according to $\mathrm{MSE}\left(x_{i}\right)$, the global historical best solution ($p_{\mathrm{gb}}$) and the individual historical best solution ($p_{\mathrm{ib}}$) are determined. Taking the *t*-th iteration as an example, if its fitness is less than the fitness corresponding to $p_{\mathrm{gb}}$, $x_{i}^{t}$ is updated as $p_{\mathrm{gb}}$. Otherwise, $p_{\mathrm{gb}}$ is not updated, as shown in Equation (18). Similarly, the update of $p_{\mathrm{ib}}$ is expressed as Equation (19). Then, the updated $p_{\mathrm{gb}}$ and $p_{\mathrm{ib}}$ are used to update the new velocity ($v_{i}$) and position ($x_{i}$) of each particle, expressed as Equations (20) and (21). Then, the updated $x_{i}$ is fed back into the ANN model as the weight and bias, and the model training is conducted again to calculate the fitness of each particle for the next iteration. Finally, when reaching the maximum iteration, the global historical best solution ($p_{\mathrm{gb}}$) is outputted as the optimal weights and bias for the ANN model to carry out the model prediction.

(18) $$p_{\mathrm{gb}}=\left\{\begin{array}{cc} x_{i}^{t}, & if\;\mathrm{MSE}\left(x_{i}^{t}\right)<\mathrm{MSE}\left(p_{\mathrm{gb}}\right)\\ p_{\mathrm{gb}}, & if\;\mathrm{MSE}\left(p_{\mathrm{gb}}\right)<\mathrm{MSE}\left(x_{i}^{t}\right) \end{array}\right.$$

(19) $$p_{\mathrm{ib}}\left(x_{i}\right)=\left\{\begin{array}{ll} x_{i}^{t}, & if\;\mathrm{MSE}\left(x_{i}^{t}\right)<\mathrm{MSE}\left(x_{i}^{t-1}\right)\\ x_{i}^{t-1}, & if\;\mathrm{MSE}\left(x_{i}^{t-1}\right)<\mathrm{MSE}\left(x_{i}^{t}\right) \end{array}\right.$$

(20) $$v_{i}^{t+1}=\omega v_{i}^{t}+c_{1}r_{1}\left[p_{\mathrm{ib}}\left(x_{i}\right)-x_{i}^{t}\right]+c_{2}r_{2}\left[p_{\mathrm{gb}}-x_{i}^{t}\right]$$

(21) $$x_{i}^{t+1}=v_{i}^{t+1}+x_{i}^{t}$$

where $p_{\mathrm{ib}}\left(x_{i}\right)$ is the individual historical best solution of the *i*-th particle. $x_{i}^{t-1}$ and $x_{i}^{t}$ are the positions of the *i*-th particle after the *t*−1 times and the *t* times of iteration, and $v_{i}^{t+1}$ and $v_{i}^{t}$ are the velocities of the *i*-th particle after the *t* + 1 times and the *t* times of iteration, respectively. *r*_1_ and *r*_2_ are random numbers between 0 and 1. *c*_1_ and *c*_2_ are the acceleration factors and are always equal to 2. *ω* represents the inertia weight, and ω=0.4~0.9.

Based on the above theory, a PSO-ANN is developed to conduct the prediction of the compressive strength of cement mortar. In PSO, the number of particles is set as 15, the iteration number is 120, and the inertia weight (ω) is 0.7 to optimize the initial weights and biases of the ANN.

### 3.5. Assessment

The determination coefficient for regression analysis (*R*^2^), the loss function MSE, and the mean absolute error (MAE) are adopted to evaluate the prediction performance of the ANN model and the effect of the intelligent optimization algorithm [56]. *R*^2^ can be used to evaluate the effectiveness of similar datasets, thereby assessing the fitting effectiveness of the model, and it is expressed as Equation (22). The MSE is used to evaluate the difference between the predicted outputs (CS) from the model and the experimental data on CS in the database. The calculation formulas for both are shown in Equation (14). The MAE, expressed as Equation (23), can quantify the average absolute differences between predicted and actual values, offering a more intuitive understanding of the average prediction deviation. When *R*^2^ = 1, MSE = 0, and MAE = 0, the predicted values of the model are accurately aligned with the experimental test values, indicating that the accuracy of the prediction model reaches 100%.

(22) $$R^{2}=1-\sum_{m=1}^{M}{\left(CS_{\mathrm{data}\text{-}m}-CS_{\mathrm{out}\text{-}m}\right)}^{2}/\sum_{m=1}^{M}{\left(CS_{\mathrm{data}\text{-}m}-{\bar{CS}}_{\mathrm{data}}\right)}^{2}$$

(23) $$\mathrm{MAE}=\frac{1}{M}\sum_{i=1}^{M}\left|CS_{\mathrm{data}\text{-}m}-CS_{\mathrm{out}\text{-}m}\right|$$

where $CS_{\mathrm{data}\text{-}m}$ is the date of CS in the database, and ${\bar{CS}}_{\mathrm{data}}$ is the average value of $CS_{\mathrm{data}\text{-}m}$.

## 4. Results and Analysis

### 4.1. Prediction Accuracy

**Figure 11:**
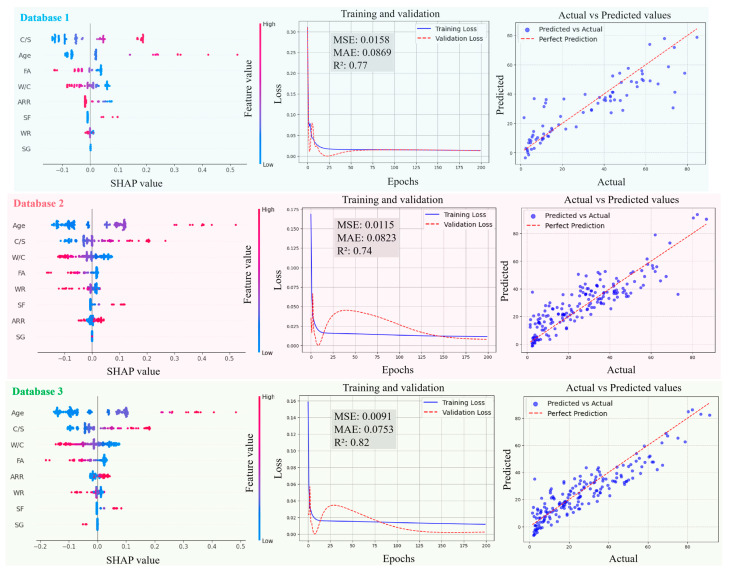
Evolution of prediction performance of ANN model in different databases.

**Figure 12:**
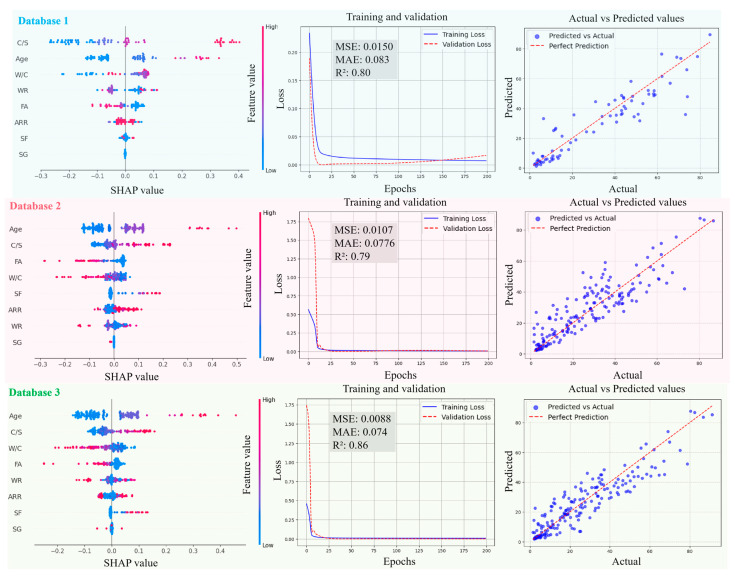
Evolution of prediction performance of GWO-ANN model in different databases.

**Figure 13:**
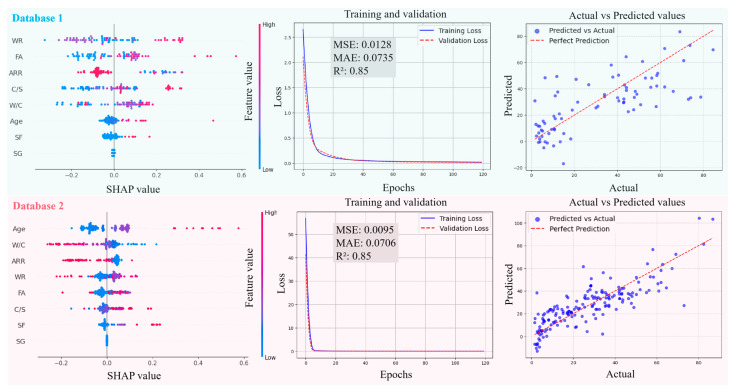

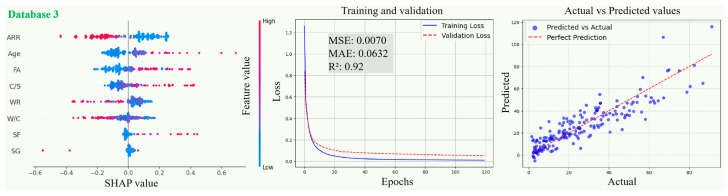
Evolution of prediction performance of PSO-ANN model in different databases.

**Figure 14:**
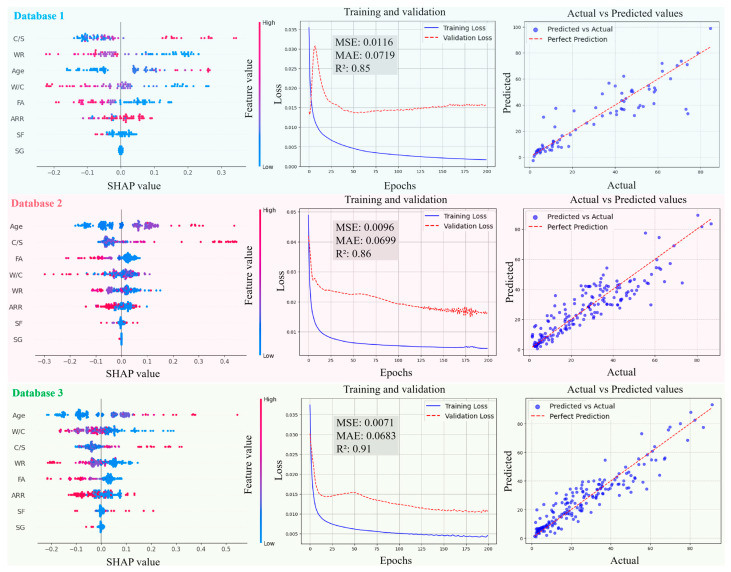
Evolution of prediction performance of GA-ANN model in different databases.

Figure 11, Figure 12, Figure 13 and Figure 14 present the SHAP interpretation plots, training and validation loss curves, and scatter plots of the predicted vs. actual values for the ANN model before and after intelligent algorithm optimization. The loss curves not only reflect the prediction accuracy of the model but also provide an intuitive view of the training process. If the training loss continues to decrease while the gap between validation and training losses widens, this indicates a potential overfitting problem, where the model learns excessively from the training data, thereby impairing its generalization ability on the validation set. Conversely, if both training and validation losses steadily decrease and converge, the risk of overfitting is low, and the model can perform well on both datasets. As shown in Figure 11, Figure 12, Figure 13 and Figure 14, the ANN, GWO-ANN, and PSO-ANN models demonstrate a favorable training status, while the GA-ANN model exhibits noticeable overfitting during training, which is primarily attributed to the inherent complexity of the GA. Due to its strong search capability, the GA tends to over-learn anomalous or non-representative data points in small datasets, such as database 1, mistakenly identifying them as critical patterns. This results in poor performance on the validation set, namely, the gap between validation and training losses widens.

**Figure 15:**
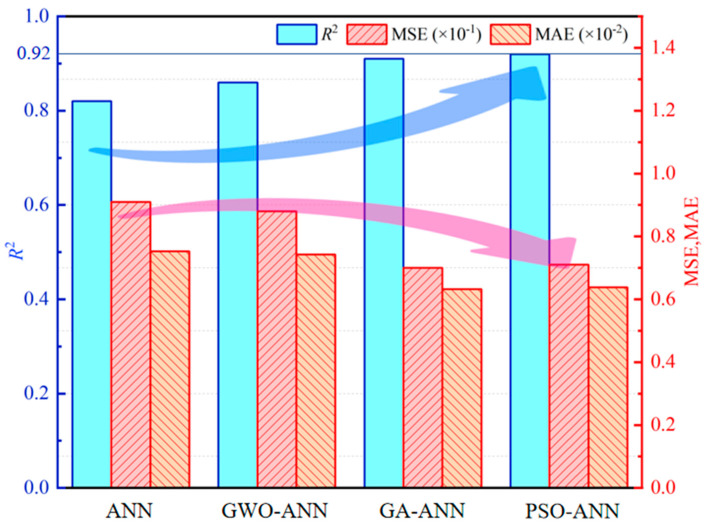
Performance evaluation comparison bar chart.

Comparing Figure 11, Figure 12, Figure 13 and Figure 14, from database 1 to database 3, the ANN model and optimized models all exhibit a consistent trend, where *R*^2^ gradually increases, while MAE and MSE decrease. This indicates that the increase in data volume and types (experimental data on RCM + OCM and model prediction data on RCM) can improve the performance indicators of the models. This is because with the increase in data samples, small-error outliers in a small database evolve into reasonable data, and larger deviations may evolve into small-error outliers, improving the accuracy of model prediction. Furthermore, the scatter plots of predicted vs. actual values in Figure 11, Figure 12, Figure 13 and Figure 14 show that, compared with the ANN model, the optimized models (GWO-ANN, PSO-ANN, and GA-ANN) produce predictions that are more tightly clustered around the regression line in different databases, further confirming their superior performance. Figure 15 further verifies this through quantitative metrics (MSE, MAE, and *R*^2^) in database 3. All optimized models (GWO-ANN, PSO-ANN, and GA-ANN) achieve significantly lower MSE and MAE values alongside higher *R*^2^ values than the standard ANN model. Notably, PSO-ANN stands out most prominently, attaining the optimal *R*^2^ (0.92) and lowest error values (MSE = 0.0070; MAE = 0.0632), providing a solid numerical foundation for the effectiveness of optimization algorithms.

The SHAP plots illustrate the distribution characteristics of SHAP values for various input variables, intuitively displaying their importance ranking and their influence (positive or negative) on the output (compression strength (CS)). In the plots, the horizontal axis represents the SHAP value, indicating the strength of its impact on the output, and the vertical axis shows the ranking of variable importance. As shown in Figure 11, Figure 12, Figure 13 and Figure 14, in the case of database 1, the W/C has the most significant impact on the compressive strength of RMC. Katz et al. [57] demonstrated that a low W/C ratio can effectively compensate for the reduction in compressive strength caused by aggregate replacement. Even at a 100% recycled aggregate replacement rate, decreasing the W/C ratio from 0.6 to 0.4 can offset the loss in compressive strength. In the experiment by Li et al. [58], the effects of reducing the aggregate packing density, lowering the water–cement ratio, and increasing the aggregate volume fraction on the improvement of RCM performance were compared. The results showed that reducing the W/C was the dominant factor in improving the mechanical property of RCM, contributing 1~2.5 times more than the other two methods. In addition, Ge et al. [59] noted that when the aggregate replacement rate (ARR) is below 30%, the strength loss of RCM with a low W/C is limited. Even under the condition of 100% ARR, the CS decreases by only about 22%. More importantly, the strategy of a low W/C can avoid the shrinkage issues of RCM, thereby enhancing its practical engineering applicability.

In the cases of database 2 and database 3, the curing age is the most influential factor affecting the CS of cement mortar. However, in the case of database 3 and the PSO-ANN model, the ARR is considered to have a more significant impact on the CS, which was demonstrated by the experimental study by Li et al. [12]. Jin et al. [60] also pointed out that as the ARR increases, the bond strength between the recycled aggregate and cement paste in RCM decreases, resulting in a decrease in the CS. The experimental results of Li et al. [14] showed that when the ARR reaches 100%, the CS of RCM decreases by 19.2% at 1 day and 12.4% at 28 days of curing, respectively. Therefore, it is recommended to limit the ARR to within 25% to achieve better performance. Accordingly, the PSO-ANN model demonstrates a better capability in identifying the influence of the ARR in database 3, aligning more closely with practical engineering observations.

In addition, in the three databases, the optimized models exhibit a significant expansion in the SHAP value distribution range, and the scatter distribution of input values becomes more dispersed, indicating a more detailed characterization of the relationships between input variables and output results. This feature validates, to some extent, the effectiveness of the GWO-ANN, PSO-ANN, and GA-ANN models in outperforming the traditional ANN model in the prediction performance of the CS of RCM.

In summary, as the number of data samples increases, the calculation accuracy of various models increases accordingly, and the order of model accuracy is PSO-BPNN≈GA-BPNN>GWO-BPNN>BPNN.

### 4.2. Prediction Error

**Figure 16:**
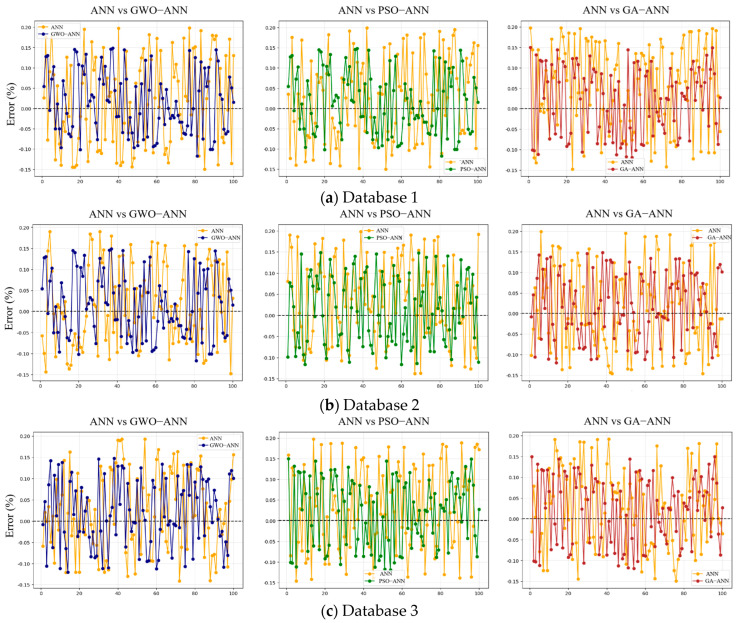
Comparison of prediction errors between ANN model and optimized models.

To further validate the predictive performance analysis of the above models, 100 data points are randomly selected from each dataset, and the prediction errors between the predicted and actual values for each model are compared, as shown in Figure 16. The prediction errors of the ANN model remain within ±20%. After introducing the GWO, PSO, and GA, the errors are further reduced and controlled within ±15%. However, the above error chart, based on randomly selected samples, only reflects the average trend of the overall error.

**Figure 17:**
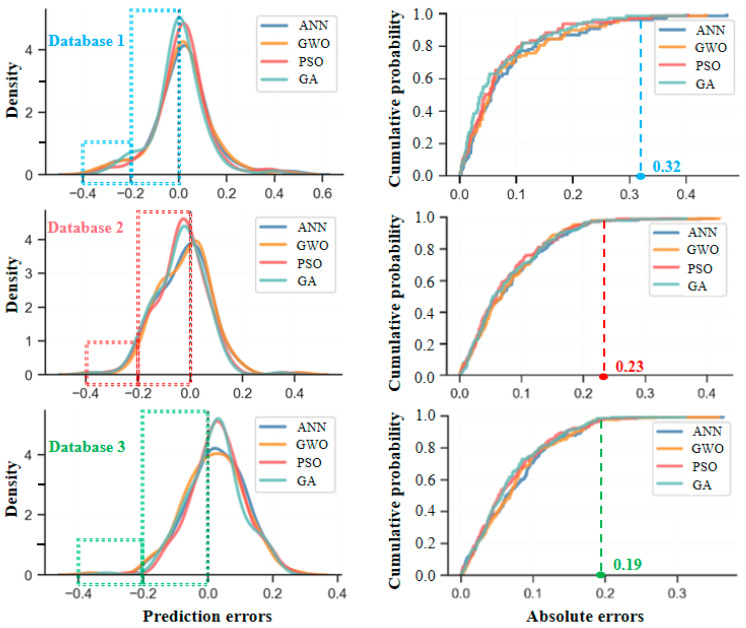
Prediction error distribution of ANN model and its optimized model.

Figure 17 gives the prediction error distribution and error accumulation of the ANN model and its optimized models in different databases. It can also be seen from this figure that for database 1, the absolute value of the prediction error is mainly concentrated within 0–20%, and a small portion of the error absolute value is in the range of 20–40%, and the individual error is beyond 40%. So, when the absolute error reaches 32%, the corresponding calculated probability is basically over 98%. Compared with database 1, the absolute error for database 2 is also mainly concentrated within 0–20%, but the density in the absolute ranges of 20–40% and 40–60% obviously reduces. For database 3, with the maximum number of data samples, the density in the absolute range of 20–40% almost tends toward zero, indicating a significant improvement in the error distribution. Notably, the error density is high near the region of ±0% and rapidly decays away from the region of ±0%, further reflecting higher stability and consistency in the model prediction.

In summary, under the condition of database 3, the calculation errors of various models are the lowest, and the order of model errors from high to low is BPNN>GWO-BPNN>PSO-BPNN≈GA-BPNN, which is consistent with the analysis of model accuracy.

**Figure 18:**
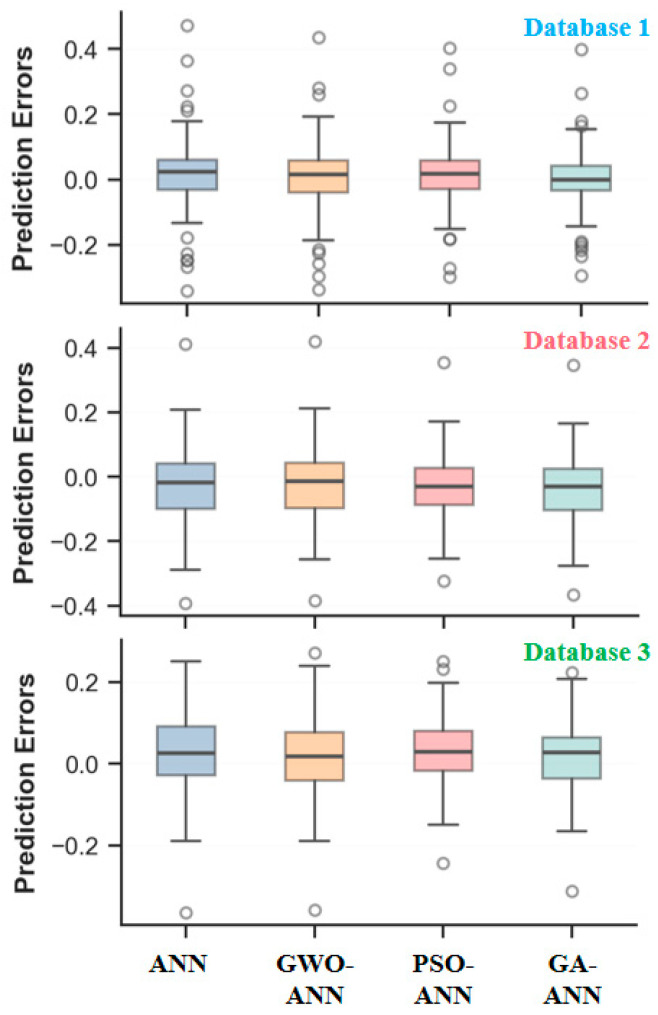
Error box diagram of ANN model and its optimized model.

Figure 18 illustrates the distribution characteristics of the prediction errors across different optimization models in different databases. The boxplots reveal that the number of outliers decreases, and their values diminish from database 1 to database 3, indicating an improvement in the prediction accuracy as the data volume increases. Concurrently, the box height (IQR, which represents the dispersion of the middle 50% of sample errors) widens, and its whiskers (reflecting the primary range of the data distribution) lengthen, suggesting a degradation in the model fitting performance. This phenomenon primarily arises because the newly incorporated data encompass a greater diversity or complexity, leading to a broader distribution of prediction errors and increased error variability. It is normal and reasonable in practical applications. In the comparative analysis of the optimization algorithms, the PSO-ANN model with the smallest box height, the fewest number of outliers, and the lowest outlier values demonstrates its superior generalization capability and stability. This advantage is primarily attributable to the exceptional global optimization capability of PSO. It efficiently explores complex, high-dimensional parameter spaces, enabling the ANN to acquire more optimal initial weights and biases. This mitigates the risk of converging to local optima, thereby facilitating a smoother and more stable learning.

### 4.3. Optimizer-Based Performance Analysis

**Figure 19:**
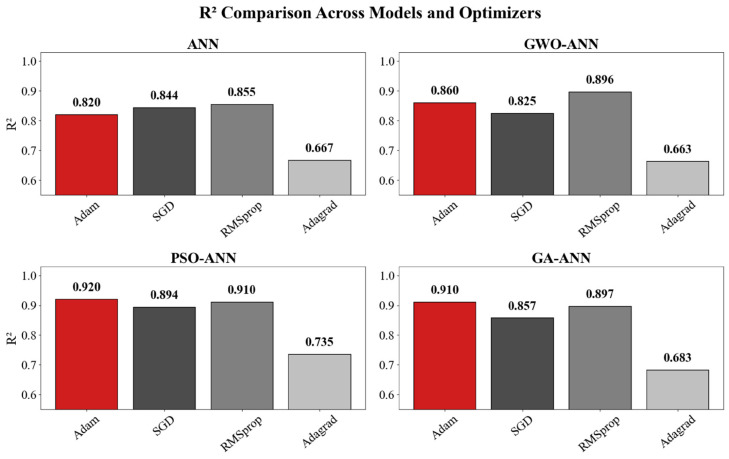
*R*^2^ of ANN models with optimizer.

**Figure 20:**
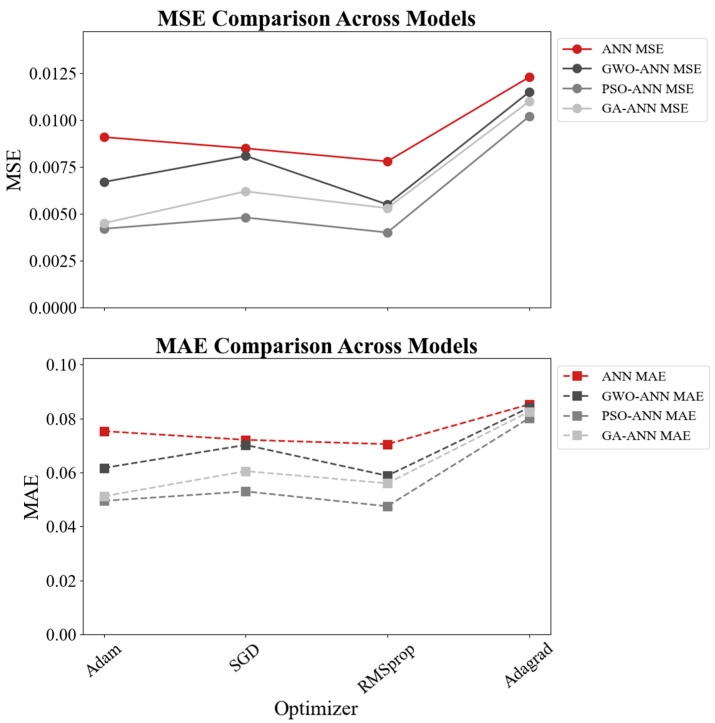
MSE and MAE comparisons.

Figure 19 and Figure 20 present the performance of the four ANN-based models (ANN, GWO-ANN, PSO-ANN, and GA-ANN) trained with four commonly used optimizers, including Adam, SGD, RMSprop, and Adagrad. Overall, the PSO-ANN model combined with the Adam optimizer demonstrates the best predictive performance among all models. Its coefficient of determination reaches 0.920, which is significantly higher than those of the baseline ANN (0.820), GWO-ANN (0.860), and GA-ANN (0.910). This indicates that the PSO algorithm effectively enhances the learning capability of the ANN and improves its ability to capture the nonlinear relationships governing the compressive strength of green recycled mortar.

In terms of error metrics, PSO-ANN with Adam achieves the lowest MSE (0.0070) and MAE (0.0594), further confirming its superiority in both accuracy and stability. Although RMSprop and SGD provide moderate improvements for certain models, the overall performance remains inferior to Adam. In contrast, Adagrad consistently exhibits the weakest predictive accuracy in all four models. For instance, its R^2^ drops to 0.66672 for the ANN and 0.663 for GWO-ANN, accompanied by significantly higher MSE and MAE values. This suggests that Adagrad is not suitable for modeling complex material behavior involving strong nonlinearity.

Taken together, the results demonstrate that the combination of PSO and the ANN, particularly when optimized using Adam, yields the most robust and reliable model for predicting the compressive strength of green recycled mortar. This finding highlights the advantage of integrating intelligent optimization algorithms with neural networks for performance prediction in sustainable construction materials.

### 4.4. Calculation Time

**Figure 21:**
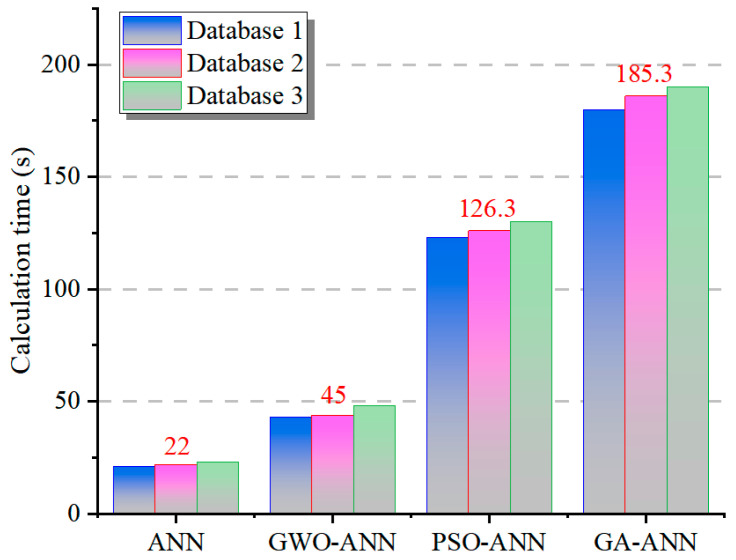
Calculation times of ANN model and optimized models in different databases.

Figure 21 illustrates the comparison of the computation times of different models in database 1 with 260 data, database 2 with 566 data, and database 3 with 611 data. It can be seen from this figure that the calculation times for the same model are very similar in different databases, and the average times for the ANN model, GWO-ANN model, PSO-ANN model, and GA-ANN model are 22 s, 45 s, 126.3 s, and 185.3 s, respectively. This means that within the range of several hundred sets of data, the amount of data does not affect the computation time of the models, while the optimization algorithms can affect the computation time. Combined with the analysis of prediction performance, its improvement is usually accompanied by a decrease in computational efficiency. The PSO-ANN model and the GA-ANN model have relatively close, excellent accuracy and are clearly superior to the GWO-ANN model, where the *R*^2^ of the former increases by 6.97% (the MAE and MSE decrease by 11.1% and 19.38%, respectively). However, in terms of computational efficiency, the PSO-ANN model is improved by 33.3% compared with the GA-ANN model. These results indicate that the PSO algorithm achieves a favorable balance between prediction accuracy and computing efficiency.

Other scholars’ research seems to confirm our viewpoint as well. In the study by Han et al. [61], the PSO-ANN model achieved the lowest values for the RMSE, MAE, and MAPE, while attaining the highest R^2^, demonstrating the strong global optimization capability of PSO. Notably, even with only 15 data used for training, the PSO-ANN model consistently exhibited the lowest RMSE and MAE, indicating a relatively low sensitivity to sample size and strong data adaptability. Additionally, the study by Li et al. [62] also validated the advantages of the PSO-ANN model in multiple performance dimensions in the prediction of cement mortar durability. Specifically, the model achieved RMSE reductions of 47%, 55%, and 53% for predicting chloride ion penetration resistance, freeze–thaw durability, and sulfate attack resistance, respectively. These results highlight the broad applicability and high accuracy of the PSO-ANN model in complex engineering prediction.

A comparative analysis of the optimization algorithms indicates that the GWO algorithm provides limited performance improvement for the ANN model. In contrast, the PSO and GA optimization algorithms significantly enhance the model’s performance on recycled mortar data, demonstrating superior prediction stability and accuracy.

## 5. Conclusions

This study integrated experimental data on RCM + OCM and model prediction data on RCM to construct three comprehensive databases with different scales. Systematic data preprocessing, feature analysis, visualization, and error assessment were subsequently performed. Leveraging these databases, ANN models coupled with three intelligent optimization algorithms, including GWO, PSO, and GA, were employed to predict the compression strength of RCM. The main results are summarized as follows.

(1) Optimization algorithms significantly enhance the predictive performance of an ANN. Compared with conventional ANNs, the hybrid models (GWO-ANN, GA-ANN, and PSO-ANN) all demonstrate superior prediction accuracy and faster convergence rates. Notably, PSO-ANN achieves optimal performance in error control (MSE = 0.007; MAE = 0.0632) and fitting capability (*R*^2^ = 0.92), followed by GA-ANN. The performance improvement offered by the GWO is relatively modest.
(2) Database scale can influence model performance. Expanding the database scale (from database 1 to databases 2 and 3) significantly improves the prediction accuracy of the models and effectively mitigates outlier issues and overfitting risks inherent in database 1 (small-sample database). This enhancement is particularly pronounced for algorithms with higher complexity (e.g., GA-ANN).
(3) Variables affect the model interpretability. Feature importance analysis and SHAP visualizations reveal scenario-dependent variations in the influence of input variables on output (compression strength). W/C, C/S, and curing age are identified as core governing variables. Optimization via GWO, PSO, and GA effectively enhances the models’ ability to discern the directional effects of input variables, while reducing inter-variable interference and error propagation.
(4) There is a trade-off between computational efficiency and predictive performance. Although intelligent optimization algorithms improve model performance (accuracy and convergence), they concurrently reduce the computational efficiency. PSO achieves a superior balance between accuracy and efficiency, reducing the training time by 33.3% compared with the GA while maintaining high predictive accuracy.

In summary, the multi-source data-driven neural network models developed for predicting the compressive strength of RCM provide robust data support and a theoretical foundation for the mixed design. Our next research work will focus on (i) expanding the diversity of experimental samples, (ii) incorporating deep learning models with more complex architectures, and (iii) developing automated mixed proportion recommendation systems.

## Figures and Tables

**Table 1:** Training dataset for machine learning model.

No.	Reference	Quantity	Proportion
1	Gao W. [29]	3	0.5
2	Li B.L. et al. [40]	9	1.5
3	Mou Y. et al. [41]	3	0.5
4	Fu Y. et al. [42]	4	0.6
5	Liu X.Y. et al. [43]	3	0.5
6	Sui Z.C. et al. [44]	81	13.2
7	Kurad R. et al. [45]	20	3.2
8	Verma S.K. et al. [46]	4	0.6
9	Dapena E. et al. [47]	18	2.9
10	Khelafi A. et al. [48]	15	2.5
11	Wu H. et al. [49]	9	1.5
12	Ning W. [1]	20	3.3
13	Li C. et al. [14]	18	3.0
14	Bamshad O. et al. [50]	36	5.9
15	Hamid, N.D. et al. [51]	270	44.2
16	Shao J.J. et al. [52]	98	16.1

**Table 2:** Data distributions of different databases.

Database	Variable	Sample Size	Maximum	Minimum	Average	25% Quantile	50% Quantile	75% Quantile
1	ARR	260	100	0	83.77	100	100	100
	W/C	260	0.99	0.2	0.56	0.365	0.52	0.75
	C/S	260	0.89	0.14	0.45	0.2	0.33	0.66
	FA	260	60	0	14.65	0	12	30
	SF	260	36	0	2.79	0	0	0
	SG	260	12	0	0.09	0	0	0
	WR	260	4	0	1.32	0.15	1.3	2.13
	Age	260	360	1	29.64	3	7	28
	CS	260	91.1	1.5	29.34	6.7	20.1	48.26
2	ARR	566	100	0	38.48	0	0	100
	W/C	566	0.99	0.2	0.46	0.3	0.44	0.5
	C/S	566	1.06	0.14	0.42	0.33	0.36	0.46
	FA	566	60	0	6.73	0	0	6
	SF	566	36	0	1.47	0	0	0
	SG	566	12	0	0.042	0	0	0
	WR	566	4.07	0	1.11	0.13	0.73	1.5
	Age	566	360	1	21.69	7	14	28
	CS	566	91.1	1.5	28.3	11.08	25.1	42.19
3	ARR	611	100	0	37.77	0	0	100
	W/C	611	0.99	0.2	0.47	0.3	0.45	0.57
	C/S	611	1.06	0.14	0.42	0.33	0.36	0.4
	FA	611	60	0	7.23	0	0	12
	SF	611	36	0	1.36	0	0	0
	SG	611	30	0	0.43	0	0	0
	WR	611	4.07	0	1.04	0.11	0.64	1.5
	Age	611	360	1	21.76	7	14	28
	CS	611	91.1	1.5	27.89	11.03	25.1	41.4

## Data Availability

The original contributions presented in this study are included in the article. Further inquiries can be directed to the corresponding author.

## Nomenclature

Acronyms	
AI	Artificial Intelligence
ARR	Aggregate Replacement Ratio
ANN	Artificial Neural Network
CNN	Convolutional Neural Network
CS	Compressive Strength
C/S	Cement–Sand Ratio
FA	Fly Ash
GA	Genetic Algorithm
GWO	Grey Wolf Optimizer
IQR	Interquartile Range
MAE	Mean Absolute Error
MSE	Mean Squared Error
OCM	Ordinary Cement Mortar
PSO	Particle Swarm Optimization
PSO-BP	PSO-Optimized Backpropagation
*R* ^2^	Coefficient of Determination
RCM	Recycled Cement Mortar
RNN	Recurrent Neural Network
RMSE	Root Mean Squared Error
SF	Silica Fume
SG	Slag
SHAP	Shapley Additive Explanations
WR	Water-Reducing Agent
W/C	Water–Cement Ratio
